# Single-Cell Transcriptomics Reveals Endothelial Plasticity During Diabetic Atherogenesis

**DOI:** 10.3389/fcell.2021.689469

**Published:** 2021-05-19

**Authors:** Guizhen Zhao, Haocheng Lu, Yuhao Liu, Yang Zhao, Tianqing Zhu, Minerva T. Garcia-Barrio, Y. Eugene Chen, Jifeng Zhang

**Affiliations:** ^1^Frankel Cardiovascular Center, Department of Internal Medicine, University of Michigan Medical Center, Ann Arbor, MI, United States; ^2^Department of Internal Medicine, The Second Xiangya Hospital, Central South University, Changsha, China

**Keywords:** single-cell RNA-sequencing, endothelial cell, transcriptomic heterogeneity, diabetes, atherosclerosis

## Abstract

Atherosclerosis is the leading cause of cardiovascular diseases, which is also the primary cause of mortality among diabetic patients. Endothelial cell (EC) dysfunction is a critical early step in the development of atherosclerosis and aggravated in the presence of concurrent diabetes. Although the heterogeneity of the organ-specific ECs has been systematically analyzed at the single-cell level in healthy conditions, their transcriptomic changes in diabetic atherosclerosis remain largely unexplored. Here, we carried out a single-cell RNA sequencing (scRNA-seq) study using EC-enriched single cells from mouse heart and aorta after 12 weeks feeding of a standard chow or a diabetogenic high-fat diet with cholesterol. We identified eight EC clusters, three of which expressed mesenchymal markers, indicative of an endothelial-to-mesenchymal transition (EndMT). Analyses of the marker genes, pathways, and biological functions revealed that ECs are highly heterogeneous and plastic both in normal and atherosclerotic conditions. The metabolic transcriptomic analysis further confirmed that EndMT-derived fibroblast-like cells are prominent in atherosclerosis, with diminished fatty acid oxidation and enhanced biological functions, including regulation of extracellular-matrix organization and apoptosis. In summary, our data characterized the phenotypic and metabolic heterogeneity of ECs in diabetes-associated atherogenesis at the single-cell level and paves the way for a deeper understanding of endothelial cell biology and EC-related cardiovascular diseases.

## Introduction

Atherosclerosis is the primary pathological basis of myocardial infarction, ischemic stroke, and peripheral vascular diseases, which represent a leading cause of death worldwide (Lusis, 2000; Herrington et al., 2016). Atherosclerosis accounts for virtually 80% of all deaths among diabetic patients (Aronson and Rayfield, 2002). Prolonged exposure to hyperglycemia is recognized as a major factor in the pathogenesis of atherosclerosis associated with diabetes. Hyperglycemia induces a large number of alterations in the vascular tissue at the cellular level that potentially accelerate the atherosclerotic process (Aronson and Rayfield, 2002; Calkin and Allen, 2006; Funk et al., 2012). Growing evidence indicates that endothelial cell (EC) dysfunction is a critical early event in the diabetes-associated development of atherosclerosis (Hansson, 2005).

Endothelial cells exist in the whole body and play an essential role in tissue homeostasis by assisting vessel formation and function, and building a barrier between blood and tissue cells (Cines et al., 1998). ECs exhibit considerable structural and functional heterogeneity depending on the tissue in which they reside, which have been demonstrated by recent single-cell RNA sequencing (scRNA-seq) studies (Feng et al., 2019; Kalluri et al., 2019; Kalucka et al., 2020; Paik et al., 2020). In cardiac development, specifically, certain ECs within the endocardium undergo a process named endothelial-mesenchymal transition (EndMT) to give rise to mesenchymal cells, which play a crucial role in endocardial cushion formation (Camenisch et al., 2002; Dejana et al., 2017). In addition, EndMT makes a major contribution to the suboptimal repair of damaged heart tissue after ischemic injury, which has also been shown through scRNA-seq (Tombor et al., 2021). Throughout life, ECs residing in the vessel wall are exposed to various mechanical stresses (Chiu and Chien, 2011; Mahmoud et al., 2017; Demos et al., 2020), inflammatory stimuli (Hajra et al., 2000; Fan et al., 2012), and metabolic alteration (Patella et al., 2015; Kuo et al., 2017; Theodorou and Boon, 2018). The extended exposure gradually leads to endothelial activation and EndMT. Recently, multiple studies extended our understanding of EndMT and demonstrated that it promotes atherosclerosis progression (Souilhol et al., 2018). Using an endothelial-lineage tracing system, EndMT-derived fibroblast-like cells were identified in mouse atherosclerotic plaques (Evrard et al., 2016). In addition to ECs undergoing EndMT to fibroblast-like cells, there may be unidentified subpopulations of ECs within the atherosclerotic lesions that remain to be explored.

The recent advent of scRNA-seq has enabled the transcriptomic analysis of a large number of cells at the single-cell resolution (Liu et al., 2019; Kalucka et al., 2020; Rohlenova et al., 2020), which provides insights into the transcriptional signature in individual cells. Here we performed scRNA-seq using endothelial-enriched single cells obtained from the mouse heart and aorta following feeding of a 12-week diabetogenic diet with cholesterol (DDC) or standard chow (Chow) diet to determine the differential gene transcription during atherosclerosis progression. Integrated analysis of scRNA-seq data revealed that ECs are heterogenous in the cardiovascular system under normal and atherosclerotic conditions. Diet-induced diabetic atherosclerosis dramatically altered EC transcriptomic profiles, including reprograming into mesenchymal cells. In addition, we identified diminished fatty acid oxidation (FAO) in the ECs undergoing mesenchymal transition in atherosclerosis, which underscore metabolic targets that control the development of atherosclerosis.

## Results

### Single-Cell RNA Sequencing Revealed Eight Endothelial Cell Clusters in the Cardiovascular System

**FIGURE 1:**
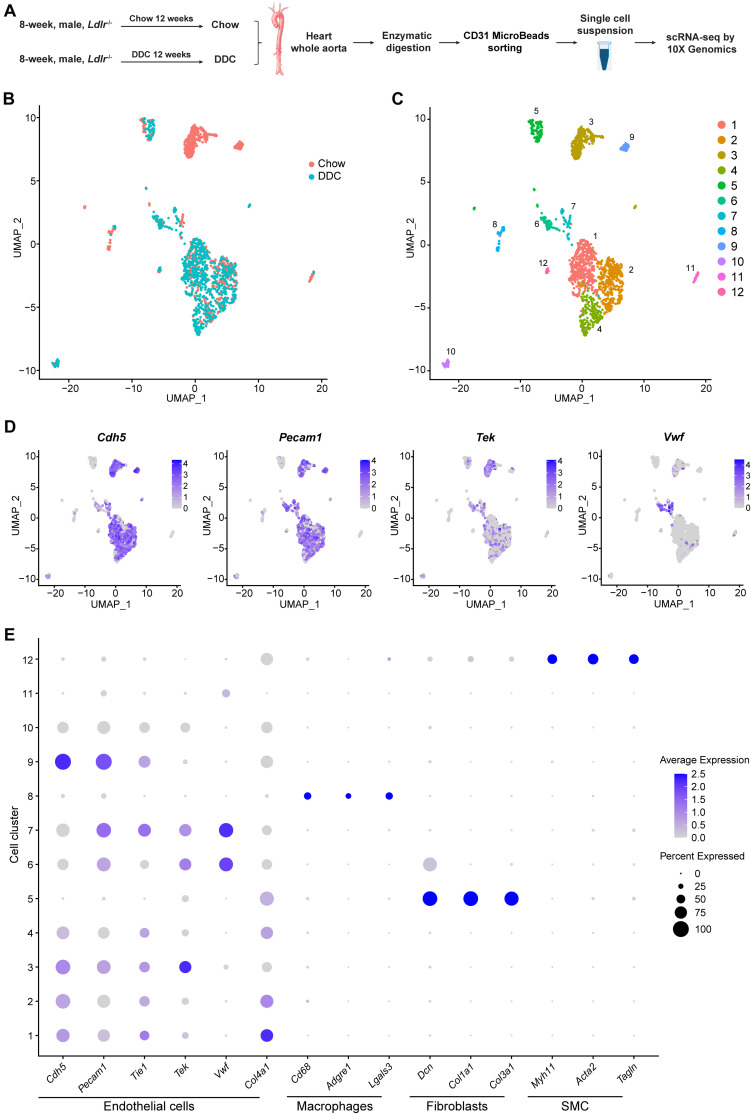
Identification of cell clusters present in the mouse heart and aorta by single-cell RNA sequencing (scRNA-seq). **(A)**, Schematic diagram indicating the procedure for scRNA-seq. Chow, *Ldlr*^–/–^ mice fed a standard chow for 12 weeks. DDC, *Ldlr*^–/–^ mice fed a diabetogenic diet with 0.15% cholesterol for 12 weeks. The cells isolated from each group were pooled from three mice. **(B)**, Uniform Manifold Approximation and Projection (UMAP) plot of aggregate cells from Chow and DDC group. Colors denote different groups. After quality control, 1014 and 770 cells from Chow and DDC were captured for clustering analysis. **(C)**, UMAP plot of aggregated cells from the three groups with colors denoting different cell clusters. **(D)**, Expression of endothelial cell (EC) marker genes (*Cdh5*, *Pecam1*, *Tek*, and *Vwf*) was visualized by Feature plots. **(E)**, Dot plot of selected marker genes for each cluster and lineage. Dot size indicates the percentage of cells expressing each gene, and dot color represents the average expression level. SMC, smooth muscle cells.

The EC heterogeneity across healthy tissues has been characterized using scRNA-seq technology (Feng et al., 2019; Kalluri et al., 2019; Kalucka et al., 2020; Paik et al., 2020). However, the EC plasticity in pathological conditions remains unexplored. To determine EC heterogeneity in diabetic atherosclerosis, we performed scRNA-seq analysis on the ECs collected from *Ldlr* null (*Ldlr*^–/–^) mice fed a diabetogenic high-fat diet with cholesterol (DDC) (Subramanian et al., 2008) or a standard Chow for 12 weeks (Figure 1A). The mice fed a DDC showed increased body weight, impaired glucose tolerance and insulin sensitivity compared to chow-fed mice (Supplementary Figures 1A–C). Additionally, a significant increase in plasma total cholesterol (TC) and triglycerides (TG) were noticed in the DDC-fed mice relative to the chow-fed mice (Supplementary Figure 1D). Next, the ECs from the heart and aorta were enzymatically isolated and purified from three mice from each group using a collagenase protocol (Hu et al., 2018). Single cells from the two groups were subsequently bar-coded and sequenced using the 10X Genomics Chromium platform (Figure 1A). The median reads per cell were 243,312 for Chow group and 276,821 for DDC group, and transcripts detected per cell were 1551 and 1297, respectively (Supplementary Table 1).

scRNA-seq data analysis was performed using Seurat version 4.0. As a quality control, we eliminated the cells that expressed gene counts >3000 or <200, and those expressing >10% mitochondrial unique molecular identifier (UMI) counts to remove doublets and damaged cells during the sample preparation. Subsequently, data integration, and unsupervised graph-based clustering were performed to group the cells according to their gene-expression profile and uniform manifold approximation and projection (UMAP) plot was used for visualization (Figure 1B). In total, 12 cell clusters were singled out in the integrated datasets (Figure 1C). To define the identity of each cell cluster, we performed differential expression analysis [by average log2(fold change)] between each cluster and all other clusters (Supplementary Table 2) and assigned a specific cell type to each cluster based on the established lineage-specific marker genes (Figures 1D,E). Next, we selected 8 EC clusters based on the expression of *Cdh5*, *Pecam1*, *Tek*, or *Vwf* (Figures 1D,E), and excluded contaminating macrophages (cluster 8) which featured expression of *Cd68*, *Adgre1*, and *Lgals3*; fibroblasts (cluster 5) which showed high expression of the collagens/collagen-binding proteins, *Dcn, Col1a1*, and *Col3a1*; and vascular smooth muscle cells (SMC, cluster 12) which highly expressed the SMC canonical markers, *Myh11*, *Acta2*, and *Tagln* (Figure 1E). Of note, the gene expression profile of cluster 11, without the expression of the canonical EC markers, could not support the lineage assignment as ECs, thus these cells were also excluded from further analysis.

### Endothelial Cell Subpopulations Show Transcriptomic and Functional Heterogeneity

**FIGURE 2:**
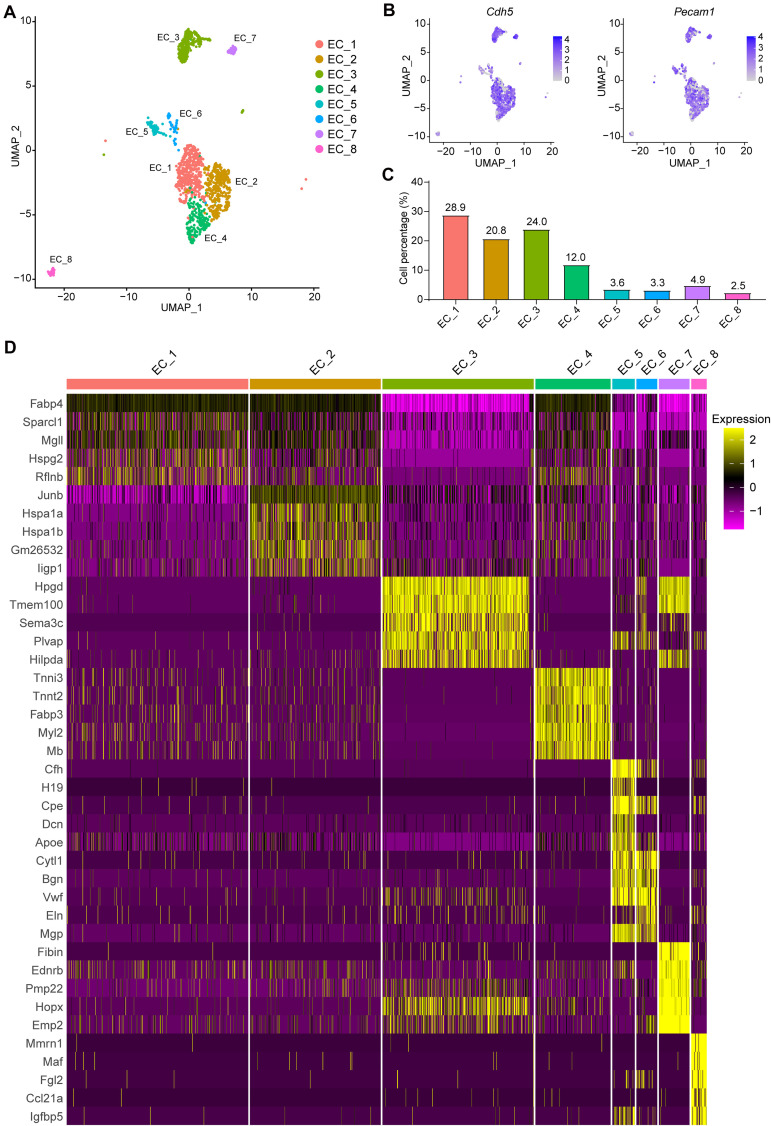
Categorization of endothelial cell subpopulations. **(A)**, UMAP plot of the aggregate ECs with positive expression of *Cdh5*, *Pecam1*, *Tek*, or *Vwf*. Eight EC clusters were identified in the aggregate arterial ECs. **(B)**, Feature plot of EC marker genes *Cdh5* and *Pecam1* in the EC clusters. **(C)**, Percentage of cells in each EC cluster. **(D)**, The top5 markers for each cluster were selected based on the average Log2(fold change). The FindAllmarkers function in Seurat v4.0 was performed using non-parametric Wilcoxon rank sum test with parameters min.pct = 0.25, thresh.use = 0.25, only.positive = TRUE and return.thresh = 0.01, to find all markers for each cluster.

**FIGURE 3:**
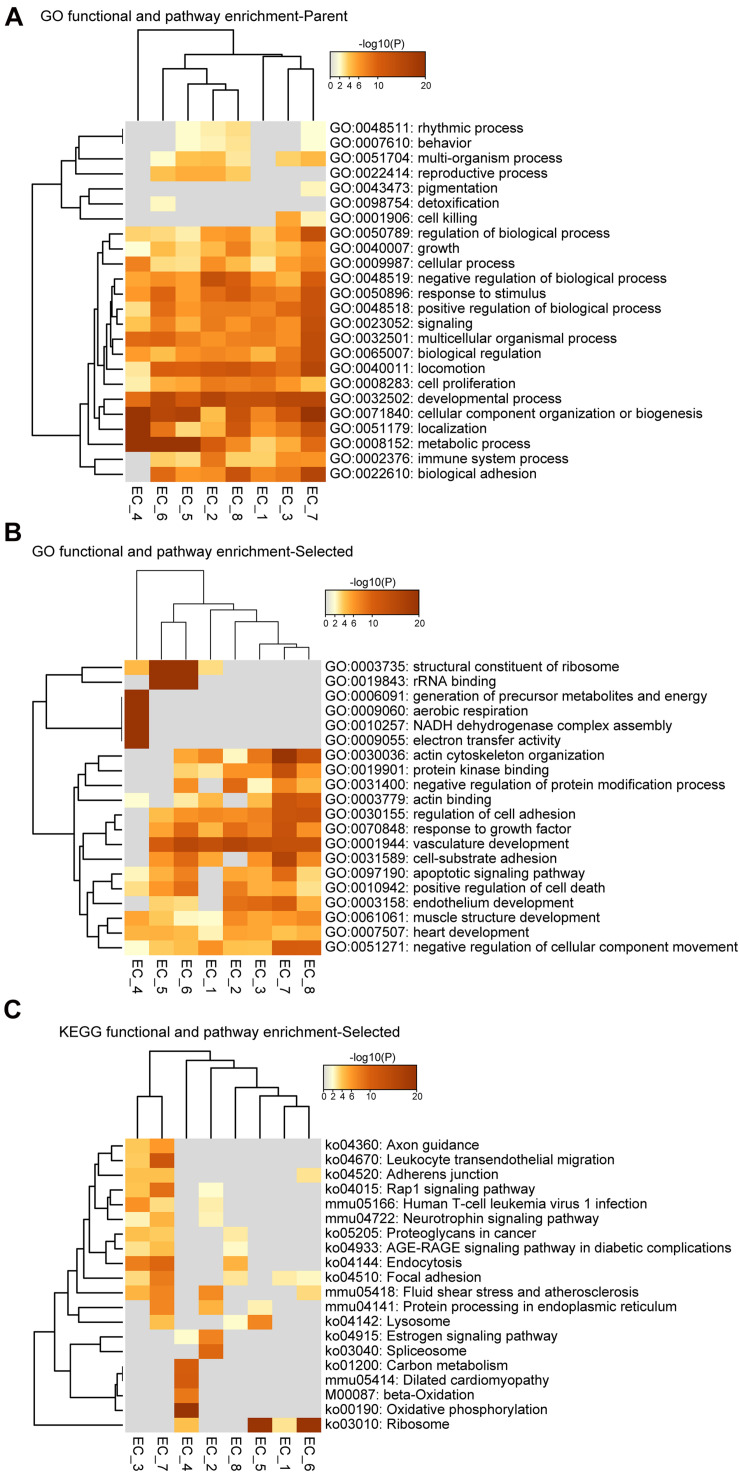
Functional and pathway enrichment analysis of endothelial cell subpopulations. **(A**–**C)**, Heatmap showing the Gene Ontology (GO) and KEGG-terms enrichment of all the cluster-specific genes for the 8 EC clusters.

Eight EC subpopulations were identified (Figure 2A) with positive expression of EC marker genes *Cdh5* and *Pecam1* (Figure 2B). Next, we sought to determine the proportion of each subpopulation, and perform differential gene expression and gene set enrichment analysis (Figures 2C, 3 and Supplementary Table 3). Notably, EC_1, EC_2, EC_3, and EC_4 are the major clusters, accounting for 85.8% of the total ECs, while the other four EC clusters (EC_5, EC_6, EC_7, and EC_8) only account for 14.2% (Figure 2C). We identified the top 5 marker genes [sorted by average log2(fold change)] for each cluster relative to all other EC clusters, and these genes were plotted using a heatmap (Supplementary Table 4 and Figure 2D). The two largest subpopulations, EC_1 and EC_2, accounted for 32 and 26.3% of all ECs, respectively, and showed high expression of genes involved in fatty acid metabolism (*Fabp4*, *Cd36*, and *MgII*) (Elmasri et al., 2009; Son et al., 2018; Theodorou and Boon, 2018) and angiogenesis markers (*Flt1* and *Kdr*) (Kanno et al., 2000; Zachary and Gliki, 2001) (Supplementary Table 3). Accordingly, the GO terms of vascular development, cell growth, and response to growth factors were enriched in these two EC clusters (Supplementary Figures 2A,B). Additionally, we found that EC_2 expressed the highest levels of the two best-known mechanosensitive genes, *Klf2*(Doddaballapur et al., 2015) and *Klf4* (Sangwung et al., 2017), and showed enrichment for the GO term regulation of cellular response to stress (Supplementary Table 3 and Supplementary Figure 2B), indicating EC_2 displaying an atheroprotective endothelial phenotype. Based on the markers specific to EC_3 (Supplementary Table 3), blood vessel morphogenesis, cell junction organization, and adherence junction organization showed selective enrichment in this cluster (Supplementary Figure 2C), suggesting its contribution to maintaining the endothelial barrier function. Interestingly, EC_4 demonstrated a high expression of *Fabp3*, *Myl2*, and *Myl3*, which have been reported to be selectively expressed in heart tissue (Andersen et al., 2012; Tsukahara et al., 2014; Sheikh et al., 2015) (Figure 2D). In line with recent scRNA-seq studies of cardiac ECs (Feng et al., 2019; Tombor et al., 2021), the GO terms, including electron transport chain, aerobic respiration, proton transmembrane transporter activity and mitochondrial electron transport, and the KEGG pathways including, cardiac muscle contraction, mitochondrial fatty acid beta-oxidation, glycolysis, and pyruvate metabolism, were enriched in EC_4 cluster (Supplementary Figure 2C), suggesting that EC_4 belongs to the cardiac lineage and exhibits distinct functional and metabolic signatures from those in the vascular ECs.

We then investigated the expression profiles enriched for EC_5, EC_6, EC_7, and EC_8. Interestingly, we found that EC_5 and EC_6 highly expressed both endothelial marker genes (*Pecam1*, *Cdh5*, and *Vwf*) and mesenchymal marker genes (Piera-Velazquez and Jimenez, 2019; Ma et al., 2020) (*Dcn*, *Mgp*, *Eln*, and *Fn1*) (Figures 2B,D and Supplementary Table 3), resembling the signature of endothelial-mesenchymal transition (EndMT). Moreover, the GO terms of vascular development, extracellular matrix organization (extracelluar matrix structural constituent for EC_5 and collagen-containing extracellular matrix for EC_6) and response to growth factor stimulus were enriched in these two EC clusters, further supporting that EC_5 and EC_6 acquire a mesenchymal state (Supplementary Figure 3A,B). Similarly, EC_8 also showed EndMT features, characterized by the expression of EC marker gene *Pecam1* and mesenchymal marker genes, including *Vim*, *Fn1*, and *Tgfbr2* (Frid et al., 2002; Derada Troletti et al., 2019; Piera-Velazquez and Jimenez, 2019) (Figure 2B and Supplementary Table 3) and are enriched for the GO term of vascular development, tissue remodeling and mesenchyme development (Supplementary Figure 3D). Additionally, EC_8 highly expressed *Fgl2* (encoded fibrinogen like protein 2), *Ccl21a*, and *Il7*, which are involved in cell adhesion and EC activation (Liu et al., 2003; Yuan et al., 2015). Unlike EC_5, EC_6, and EC_8, the EC_7 cluster selectively expresses genes associated with actin cytoskeleton organization, blood vessel morphogenesis, cell junction organization, and endothelium development, suggesting that this EC cluster may be involved in maintaining endothelial function (Supplementary Figure 3C).

To further identify the cellular functions of each EC cluster, we performed GO and KEGG enrichment analysis of the gene sets of these clusters (Figure 3). We found that all EC clusters shared the GO terms associated with the developmental process, cellular component organization or biogenesis, and metabolic process (Figure 3A). The vascular development pathway demonstrated selective enrichment in all EC clusters except EC_4 (Figure 3B), further supporting its endocardial lineage assignment which is distinct from the other seven EC clusters. Additionally, as shown in Figures 3B,C, we found that EC_4 showed enrichment of NADH dehydrogenase activity, electron transfer activity, beta-oxidation, carbon metabolism, and oxidative phosphorylation, which is in line with previous reports that endocardial ECs have high metabolic demands to execute their biological functions in the high-energy-requiring heart (Eelen et al., 2015; Xiong et al., 2018; Shimizu et al., 2020).

### Identification of Endothelial Cell Plasticity in Diabetic Atherosclerosis

**FIGURE 4:**
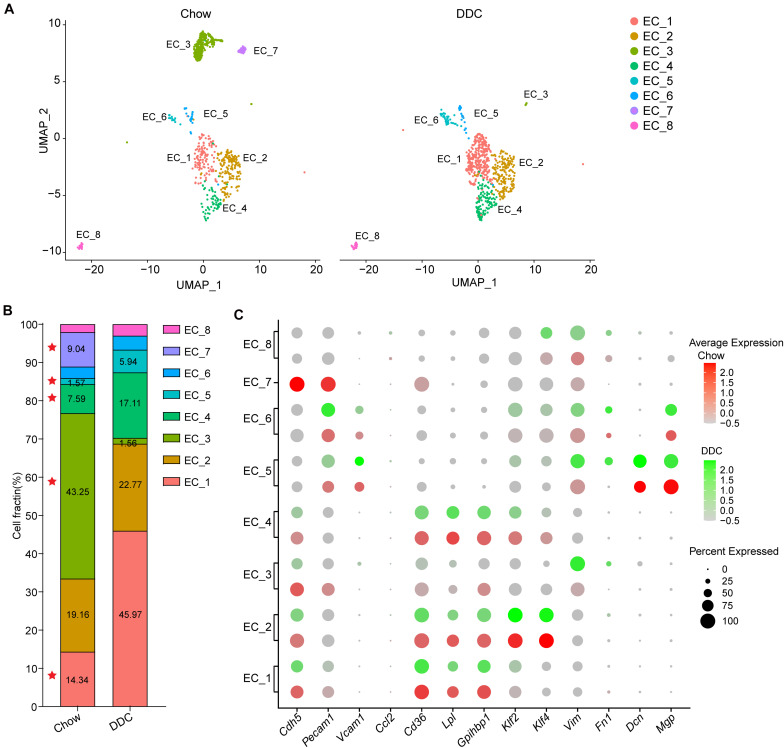
Diet-induced changes in endothelial cell subpopulations. **(A)**, UMAP plot of EC clusters from Chow (*n* = 830) and DDC (*n* = 707) groups (n is the number of cells qualified for analysis in each group). Colors denote different EC clusters. **(B)**, Cell cluster percentages across two experimental groups (Chow and DDC). **(C)**, Dot plot showing the expression of EC marker genes and genes associated with inflammation, lipid metabolism, stress response and endothelial-mesenchymal transition (EndMT) in all EC clusters across the two experimental conditions.

To determine the cellular response of the EC subpopulations during atherosclerosis at the single-cell level, we analyzed the scRNA-seq profiles from Chow and DDC groups (Figure 4A). Compared with the Chow group, DDC feeding resulted in expansions of EC_1, EC_4, and EC_5 subpopulations and significantly decreased EC_3 and EC_7 subpopulations (Figure 4B). There is no discernable difference between the two groups in EC_6 and EC_8 subpopulations. Compared with other EC clusters, EC_1 showed a high expression of lipid-handling genes, including *Cd36*, *Lpl*, and *Gpihbp1*, suggesting a functional specialization in lipid uptake and metabolism (Figure 4C and Supplementary Figure 4A). Accordingly, the PPAR signaling pathway was also enriched in the EC_1 cluster (Supplementary Figure 2A), in line with the master role of PPAR signaling in regulating lipid-handling genes and circulating fatty acids (Kanda et al., 2009; Mehrotra et al., 2014). Similarly, EC_4 highly expressed *Cd36* (Son et al., 2018), *Lpl* (Wang et al., 2007), and *Gpihbp1* (Chiu Amy et al., 2016) (Figure 4C and Supplementary Figure 4A), since cardiac tissues are highly oxidative and catabolize fatty acids as a source of energy under normal conditions (Schoonderwoerd and Stam, 1992; Son et al., 2018). Interestingly, DDC induced expansions of these two clusters, but did not affect the expression of lipid-handling genes (Figures 4B,C and Supplementary Figure 4A). Desipe not transforming to foam cells or accumulating cholesterol as macrophages or SMCs in the atherosclerotic plaques, ECs express transporters for cholesterol and have the biochemical pathways for cholesterol homeostasis (Hassan et al., 2006). Mutiple proteins, such as ATP-binding cassette transporter A1 (ABCA1), ABC G subfamily (ABCG1), and SR-B1 (encoded by *Scarb1*) are involved in endothelial cholesterol homeostasis (Hassan et al., 2006; Stamatikos et al., 2019). Of note, *Abca1*, *Abcg1*, and *Scarb1* showed heterogeneous expression by EC clusters (Figure 4C and Supplementary Figure 4A). *Abca1* was expressed in EC_5 and EC_8, while *Abcg1* and *Scarb1* expressed in EC_7, consistent with previous reports that ABCA1 only weakly expressed in human vascular ECs (Hassan et al., 2006). These data suggest the functional heterogeneity of cholesterol metabolism in individual EC clusters. Pathological conditions, including hyperlipidemia, hyperglycemia, disturbed flow, and vascular inflammation, lead to endothelial activation and EndMT (Piera-Velazquez and Jimenez, 2019). Consistently, we found that EC_5 showed high expression of the pro-inflammatory gene *Vcam1*, and mesenchymal marker genes *Vim*, *Fn1*, *Dcn*, and *Mgp*, and these genes were increased in the DDC group compared to the Chow group (Figure 4C and Supplementary Figure 4B), suggesting that mesenchymal activation contributes to the expansion of this cluster of ECs. Of note, different from the expression profiles of *Fn1*, *Dcn*, and *Mgp*, *Vim* was broadly expressed in all EC clusters albeit highly expressed in EC_5, EC_6, and EC_8, particularly in DDC condition, suggesting EndMT is a process in which loss of EC markers and gain of multiple mesenchymal markers. Compared to the other subpopulations, EC_3 and EC_7 showed high expression of the canonical EC markers, but low expression of pro-inflammatory genes, lipid-handling genes, and mesenchymal marker genes (e.g., *Fn1*, *Dcn*, and *Mgp*) (Figure 4C and Supplementary Figure 4B). In addition, the proportion of these two clusters were dramatically decreased after DDC feeding. Notably, the EC_7 cluster accounts for 9.04% in the Chow group, and they disappeared upon DDC feeding (Figure 4B). These data indicate that the reduction of EC_3 and EC_7 may be caused by EC apoptosis or EndMT, both occurring during atherogenesis.

### Identification of Endothelial to Mesenchymal Transition During Atherosclerosis

**FIGURE 5:**
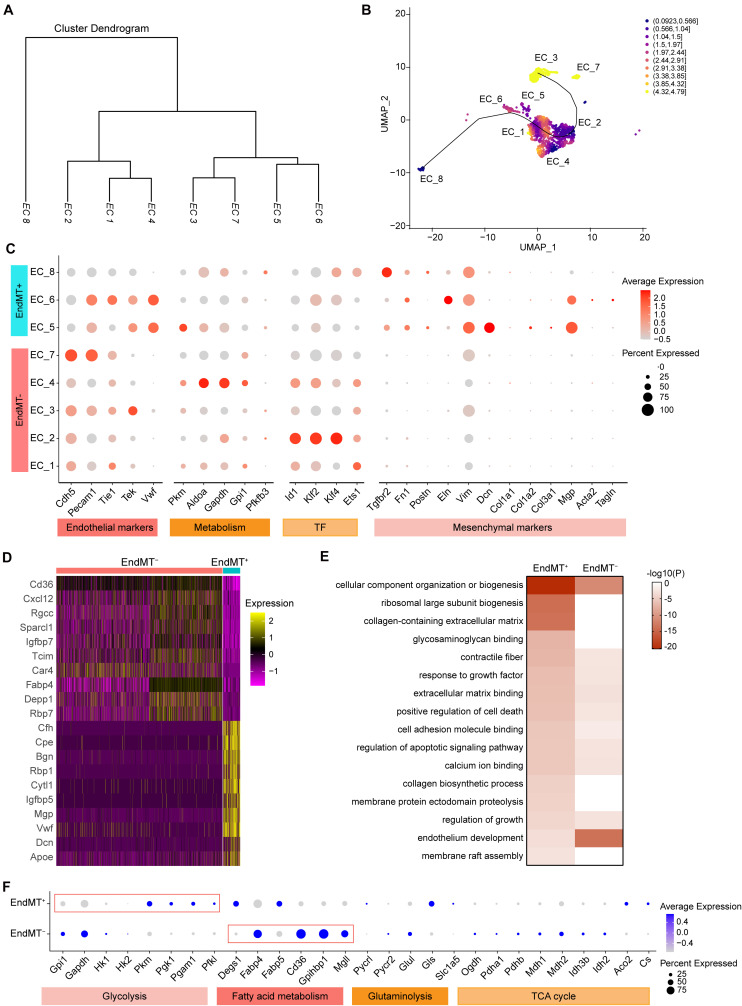
Identification of endothelial cell subpopulations undergoing mesenchymal transition. **(A)**, Dendrogram summarizing the similarity of the EC clusters in the aggregate arterial ECs according to average RNA expression. **(B)**, Two-dimensional representation of EC clusters using UMAP, with trajectory inferred by the interpretation of Slingshot and tradeSeq analysis. Color indicates imbalance score. Regions with a high score indicate that the local cell distribution is unbalanced compared to the overall distribution. **(C)**, Dot plot showing the marker genes associated with EC identity, metabolism, transcription factor, and mesenchymal identity in EndMT^–^ (EC marker positive and mesenchymal marker negative) and EndMT^+^ (EC marker positive and mesenchymal marker positive). Size depicts the percentage of cell expressing each gene, and color indicates the expression level. **(D)**, Heatmap showing the top10 genes selected from all markers of EndMT^–^ and EndMT^+^ cells based on the average Log2(fold change). **(E)**, Heatmap showing the representative GO-terms enrichment of the specific genes in EndMT^+^ cells in comparison to EndMT^–^ cells. The color indicates the -log_10_(P) value. **(F)**, Dot plot showing the expression of genes associated with glycolysis, fatty acid metabolism, glutaminolysis, and tricarboxylic acid (TCA) cycle comparing EndMT^–^ and EndMT^+^ cells.

Our analysis of the transcriptomic and functional profiles for each EC subpopulation suggests that ECs undergo phenotypic changes and metabolic programming during the development of atherosclerosis. The cluster dendrogram in Figure 5A suggests that EC_1, EC_2, and EC_4 are more similar to each other. The similarity between EC_3 and EC_7, and the similarity between EC_5 and EC_6 are also shown in Figure 5A. We next sought to explore if ECs underwent a trans-differentiation trajectory during atherogenesis and if EC trans-differentiation was associated with metabolic-related transcriptomic changes. Thus, we used Slingshot to perform trajectory inference analysis of the ECs from the two experimental groups. EC_8, EC_5, EC_6, EC_1, and EC_2 clusters were distributed in the global path along the development axis, predicting the potential hierarchy between mesenchymal clusters and mature EC clusters (Figure 5B). This prediction provides further evidence that EndMT contributes to atherosclerosis, as previously established by lineage tracing (Evrard et al., 2016). To further characterize the ECs undergoing EndMT, we compared the transcriptome of ECs that express mesenchymal markers, to ECs lacking those marker genes. As expected, the EndMT cells (EndMT^+^), including EC_5, EC_6, and EC_8, highly expressed mesenchymal and extracellular matrix genes, such as *Tgfbr2*, *Fn1*, *Eln*, *Vim*, *Dcn*, and *Mgp* (Figures 5C,D and Supplementary Figure 4B). In contrast, EC clusters, including EC_1, EC_2, EC_3, EC_4, and EC_7, that did not undergo EndMT (EndMT^–^) showed high expression of genes involved in glycolysis (*Aldoa*, *Gapdh*, *Gpi1*, and *Pfkfb3*), fatty acid metabolism (*Cd36* and *Fabp4*), vasculoprotection (*Klf2* and *Klf4*) and angiogenic response (*Id1* and *Ets1*) (Figures 5C,D and Supplementary Figure 4A). Next, the specific genes found in EndMT^+^ cells when compared with EndMT^–^ cells (Supplementary Table 5) were subjected to GO analysis. As shown in Figure 5E, EndMT^+^ cells exhibited induction of many well-known biological processes associated with proatherogenic pathways, including extracellular matrix organization, apoptosis, and adhesion molecule binding (Figure 5E). These results suggest that the ECs in a mesenchymal state show a pro-atherogenic phenotype.

Since EC metabolic reprogramming is known to play a critical role in endothelial plasticity and EndMT (Rohlenova et al., 2020), we performed a detailed analysis of the gene expression signatures related to glycolysis, fatty acid and glutamine metabolism, as well as TCA cycle (Figure 5F). In normal ECs, glycolysis is the main energy supplier, accounting for > 85% of the total ATP production (Krützfeldt et al., 1990; De Bock et al., 2013), which safeguards ECs against oxidative stress in oxygen-replete. Interestingly, EndMT^+^ cells showed reduced expression of glycolytic enzyme genes, including *Gpi1* and *Gapdh*, while increased expression of *Pkm*, *Pgk1*, *Pgam1*, and *Pfkl*, compared to EndMT^–^ cells, suggesting a glycolytic reprogramming during EndMT. Although ECs rely primarily on glycolysis as their ATP source, some studies demonstrated that fatty acid oxidation (FAO) is required to maintain EC fate and restrain EndMT (Schoors et al., 2015; Xiong et al., 2018). Consistently, EndMT^+^ cells displayed reduced expression of genes associated with fatty acid metabolism, including *Fabp4*, *Cd36*, *Gpihbp1*, and *Mgll*, further supporting that FAO inhibition potentiates EndMT (Xiong et al., 2018).

### Endothelial to Mesenchymal Transition Is Increased in Atherosclerosis

**FIGURE 6:**
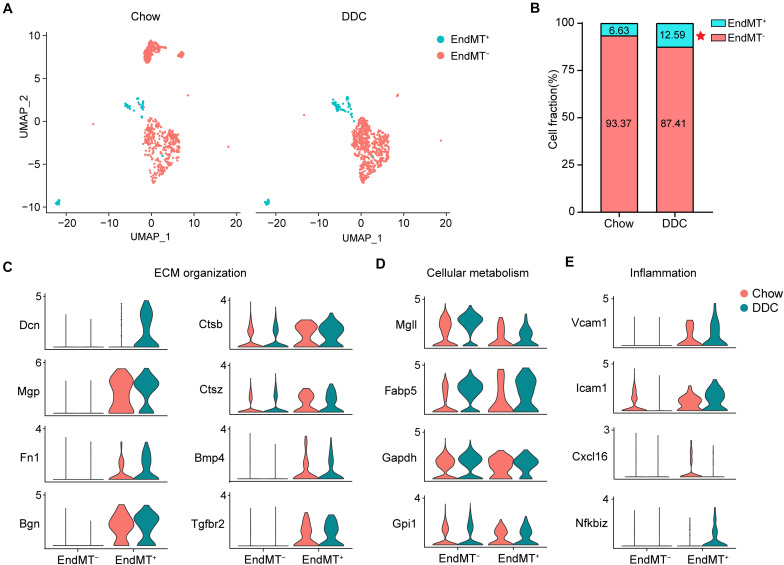
Activated endothelial cells show distinct transcriptional features. **(A)**, UMAP plot of EndMT^–^ and EndMT^+^ cells across the two experimental groups. **(B)**, The percentage of EndMT^–^ and EndMT^+^ cells across the two experimental groups. *P < 0.05 using Chi-square test. **(C**–**E)**, Violin plot showing the genes associated with ECM organization **(C)**, cellular metabolism **(D)**, and inflammation **(E)** in EndMT^–^ and EndMT^+^ cells across the two experimental groups.

The contribution of EndMT to atherosclerosis progression has been documented using endothelial lineage tracking in a mouse atherosclerosis model (Evrard et al., 2016). Consistently, our data showed that DDC markedly increased the proportion of EndMT^+^ cells relative to the Chow group (DDC, 12.59% vs. Chow, 6.63%) (Figures 6A,B). EndMT is characterized by the loss of EC features and acquisition of mesenchymal characteristics, including enhanced extracellular matrix (ECM) organization (Dahal et al., 2017), metabolic program (Xiong et al., 2018), and enhanced inflammation (Chen et al., 2015). Next, we further analyzed the alteration of the genes associated with those EndMT characteristics between the two experimental groups. As shown in Figure 6C, the ECM genes, such as *Dcn*, *Mgp*, *Fn1*, and *Bgn* were markedly increased in EndMT^+^ cell, and DDC feeding further enhanced the expression of *Dcn* and *Fn1*, in line with a prior study identifying that ECs transform toward a mature fibroblast-like phenotype during atherosclerosis progression (Souilhol et al., 2018). Additionally, EndMT^+^ cells showed increased expression of *Ctsb*, *Ctsz*, *Bmp4*, and *Tgfbr2*, all involved in ECM organization, and the ECM degradation enzymes *Ctsb* and *Ctsz* were further increased upon DDC feeding (Figure 6C), suggesting that DDC diet may induce plaque instability. To further address the metabolic program during EndMT between Chow and DDC groups, we analyzed the marker genes of fatty acid metabolism (*Mgll* and *Fabp5*) and glycolysis (*Gapdh* and *Gpi1*). We found that DDC feeding increased the cells expressing *Mgll* and *Fabp5*, in both the EndMT^–^ and EndMT^+^ cells, despite no alterations of the expression levels (Figure 6D). Moreover, the pro-inflammatory genes (*Vcam1*, *Icam1*, and *Cxcl16*) and the NF-κB pathway gene (*Nfkbiz*) were highly expressed in the EndMT^+^ cells, and DDC feeding further increased the expression of *Vcam1*, *Icam1*, and *Nfkbiz*, suggesting DDC enhances inflammatory response during EndMT (Figure 6E). These data suggest that EndMT occurs in atherosclerosis and that DDC feeding enhances the EndMT process.

## Discussion

Endothelial cell heterogeneity is a hallmark of blood vessel biology, with subpopulations identified across different tissues and within a particular vascular bed to meet the distinct physiological needs (Feng et al., 2019; Kalucka et al., 2020; Paik et al., 2020). The scRNA-seq technology has enabled the unbiased and systematic analysis of the transcriptomes of various cell populations at the single-cell resolution at once. Herein, we performed a detailed analysis, via scRNA-seq, of the cardiac and aortic EC gene expression signatures during atherosclerosis. We identified 8 EC populations in the normal tissues and characterized the heterogeneity of the transcriptome and biological process signatures of each population. Furthermore, through the integrated analysis of the ECs across normal and atherosclerotic conditions, we identified three EC clusters undergoing EndMT to a fibroblast-like phenotype, which were expanded in atherosclerosis and exhibited a metabolic switch compared with the other EC subpopulations.

It was reported that EndMT-derived fibroblast-like cells contribute to the atherosclerotic plaques, using the endothelial-lineage tracing mouse model (Evrard et al., 2016). In our study, 12.59% of ECs acquire a fibroblast phenotype in atherosclerotic conditions, which is much lower than the extent of EndMT-derived fibroblast-like cells (22.8%) in the lineage tracing study. Nevertheless, among the entire EC-derived fibroblasts, 11.1% of cells co-expressed endothelial and fibroblast markers, which is closer to the proportion in our study (12.59%). The unbiased nature of the present study further strengthens those previous findings. However, as a limitation, our study was performed using the CD31-enriched ECs, therefore, the ECs that translate toward a more mature fibroblast phenotype and lose the endothelial markers cannot be captured in the present study. Furthermore, both our study and the endothelial-lineage tracing study similarly demonstrated that ECs which undergo EndMT rarely give rise to vascular smooth muscle cells (VSMCs) in plaques. However, some studies showed that VSMCs or immune cells arise from EndMT in angiogenesis (Rohlenova et al., 2020) or in carotid artery exposed to disturbed flow (Andueza et al., 2020). Unexpectedly, we also identified the presence of EndMT-derived fibroblast-like cells in the Chow group (6.63% EndMT^+^ of total ECs). Similarly, a recent scRNA-seq study revealed the presence of angiogenic and proliferating ECs in healthy tissues (Kalucka et al., 2020). Although limited EC proliferative potential has been described in the suboptimal repair of damaged heart tissue after ischemic injury, whether these activated/proliferative ECs in the healthy tissues represent an ongoing EC repair/regeneration process remains to be further determined. Collectively, these data strengthen the evidences of phenotypic heterogeneity of ECs residing in different tissues and response to environmental conditions.

An additional level of EC heterogeneity was observed when analyzing GO biological processes for distinct EC populations. The EndMT-derived fibroblast-like cells show signatures of genes associated with extracellular matrix organization, cell apoptosis and cytokine production, which are representative key functions for fibroblasts in atherosclerosis (Brokopp et al., 2011; Evrard et al., 2016). Moreover, our findings defined that fibroblasts are the primary mesenchymal cell type derived from EndMT in atherosclerosis, which is consistent with previous reports (Evrard et al., 2016; Wesseling et al., 2018). Although the functional importance of EndMT in atherosclerotic plaque formation has been well-established (Chen et al., 2015; Evrard et al., 2016; Souilhol et al., 2018; Piera-Velazquez and Jimenez, 2019), the transcriptomic and functional heterogeneity of mesenchymal cells in atherosclerosis has yet to be elucidated. Here we identified three clusters of EndMT^+^ cells and characterized their distinct gene profiles and functional characteristics, which extend our understanding of the mesenchymal heterogeneity in atherosclerotic plaques. A recent report (Tombor et al., 2021) suggests that the EndMT in the myocardial infarction area is a reversible process, and that inhibition of TGF-β signaling could reduce the expansion of these cells. Moreover, inhibition of EndMT driving transcription factors such as *Snail* and *Slug*, has been shown to prevent EC migration and *in vitro* angiogenesis (Welch-Reardon et al., 2014; Hultgren et al., 2020). However, more studies and deeper analysis will be needed to investigate the implications of these observations in the EndMT process in atherosclerosis.

As another phenotypic readout of our scRNA-seq analysis, we also characterized the metabolic transcriptome signatures that differ between EndMT positive and negative ECs. In agreement with the observation that metabolic adaptations of ECs contribute to endothelial plasticity and EndMT in response to various physiological and pathological stimuli (Eelen et al., 2015; Theodorou and Boon, 2018; Rohlenova et al., 2020; Tombor et al., 2021), the immature ECs, which express mesenchymal markers, have a more robust glycolytic gene signature than the mature ECs. In turn, mature ECs highly express genes associated with fatty acid signaling and the TCA cycle despite still generating >85% of their ATP glycolytically. During the process of atherosclerosis, pro-inflammatory cytokines enhance glycolysis in ECs, and in turn enhanced glycolysis can drive pro-inflammatory programs, thereby constituting a vicious cycle resulting in sustained pro-inflammatory signaling in ECs (Theodorou and Boon, 2018). Here, we identified increased expression of glycolytic enzymes during EndMT. In agreement with a previous study (Xiong et al., 2018), our scRNA-seq data also revealed that EndMT in ECs is accompanied with an inhibition of FAO, suggesting that endothelial FAO may be a critical regulator of the EndMT process. Metabolic adaptations were also identified in cardiac ECs after myocardial infarction (Tombor et al., 2021) and ECs in choroidal neovascularization (Rohlenova et al., 2020). These findings further illustrate the extensive phenotypic plasticity and potential of ECs to adapt their metabolic signatures, presumably to fine-tune their function in different vascular compartments and in response to physiological and pathological conditions.

As the pump of the circulatory system, the heart has multiple types of ECs, including endocardial ECs, coronary vascular ECs, and aorta-specific ECs. Recent scRNA-seq studies have identified the transcriptome signatures of these ECs (Feng et al., 2019). Through gene expression analysis, *Cytl1* and *Npr3* were reported to be expressed in endocardial ECs, while *Fabp4* and *Cd36* were identified as the markers of coronary vascular ECs, and *Edh3* and *Fam167b* as the top 2 genes specifically expressed in aorta-specific ECs. Although our study was not designed to explore the transcriptomic differences in the organ-specific ECs, we were able to identify a cluster of cardiac ECs, EC_4, which distinguish themselves from those in other organs by specifically expressing genes involved in electron transport, cardiac muscle contraction, pyruvate and lipid metabolism. However, the cell lineage of the cardiac EC cluster singled out in our study was not able to be identified based on the markers featured in the three types of cardiac ECs reported in that prior study.

In summary, we report an unbiased single-cell transcriptomic approach to delineate the heterogeneity of cardiovascular ECs from normal and diabetic atherosclerotic conditions in mice. The identification of three ECs that very strongly recapitulate endothelial-derived fibroblasts, clearly confirms the existence of cells expressing mesenchymal markers at the single-cell level, and therefore supports the many studies reporting EndMT in atherosclerosis and other cardiovascular diseases. We also characterized the metabolic heterogeneity of ECs, which may serve as a valuable source for researchers in the field. The insights provided by our data can lead to a better understanding of endothelial biology and pathobiology and further facilitate the development of therapeutics for atherosclerosis.

## Materials and Methods

### Animals and Diet

The B6.129S7-*Ldlr*^*tm1Her*^/J (*Ldlr*^–/–^, stock No. 002207) mice were purchased from The Jackson Laboratory (Bar Harbor, ME). Eight-week-old male *Ldlr*^–/–^ mice were fed either a standard rodent chow (Chow, LabDiet 5L0D, 13% fat) or a diabetogenic diet with 0.15% cholesterol (Subramanian et al., 2008) (DDC; Envigo, TD.180368, a diet formulated with 59.1% kcal from fat and 24% sucrose with 0.15% cholesterol added) for 12 weeks. The diabetogenic diet provides 35.7% calories from fat and 37.8% from carbohydrates. Animals were housed in cages with microisolator filter tops, maintained on a 12-h light/dark cycle in a temperature-controlled room, and given free access to food and water. All animal care and experimental procedures were approved by the Institutional Animal Care & Use Committee (IACUC) from the University of Michigan and complied with the National Institutes of Health (NIH) Guidelines for the care and use of laboratory animals.

### Oral Glucose Tolerance (OGTT) and Insulin Tolerance Test (ITT)

For OGTT, mice on standard chow or DDC were fasted overnight. Next, glucose was administered (2g/kg, gavage) and blood glucose levels were measured at the indicated time points thereafter. For ITT, mice were administered human insulin (Humulin R, 1 U/kg, i.p.) following 6 h of fasting. Blood glucose levels were measured at indicated time points after insulin administration.

### Endothelial Cell Preparation

At the end of the experiments, mice were anesthetized by using carbon dioxide (CO_2_) in accordance with the NIH Guidelines for the euthanasia of animals and then perfused with 10 ml of PBS through left ventricular puncture. Preparation of the single-cell suspension of the arterial endothelial cells was performed as previously described (Hu et al., 2018). In brief, the heart and whole aorta from three mice for each group were harvested and dissected into small pieces. Samples were incubated in digestion solution [DMEM (ThermoFisher Scientific, 21063-045), 2mg/ml collagenase I (Worthington Biochemical Corporation, LS004196), 60 U/ml DNase I (Roche Diagnostics, 1010459001)] at 37°C for 30–45 min with shaking. At the end of incubation, DMEM containing 10% FBS was added to the samples to stop the digestion and filtered through a 40 μm strainer. The cell suspension was centrifuged at 400 g for 8 min at 4°C, and the pellet was re-suspended in a DPBS-based wash buffer (WB, containing 1% FBS and 2 mM EDTA). Next, the cell suspension was enriched for ECs using CD31 MicroBeads (Miltenyi Biotec, 130097418) according to the manufacturer’s instructions. The CD31-enriched single-cell suspension was washed with WB and stained with Viable dye eFluor 450 (Thermo Fisher Scientific, 50-112-8817). Doublets were gated out prior to FACS sorting, and the viable cells were sorted into a collecting tube with DMEM containing 20% FBS.

### Single-Cell RNA Sequencing (scRNA-seq)

Standard 10× Chromium Single Cell 3’ Solution v3.1 (10X Genomics Gemcode Technology) protocols were followed for scRNA-seq. Briefly, single cells with specific 10X Barcode Gel Beads and unique molecular identifiers were partitioned into the Gel Bead-in-Emulsion (GEM) in the GemCode instrument, where cell lysis and bar-coded reverse transcription of RNA ensued, followed by amplification, shearing, and 5’ adaptor and sample index attachment. Libraries were sequenced on the sequencing platform Illumina NovaSeq 6000. The sequencing depths among the samples were: 243,312 mean reads per cell for the Chow group; 276,821 mean reads per cell for the DDC group. The fraction reads in cells for each sample were >85%. The median number of genes detected per cell (also named as median genes per cell) for Chow and DDC were 1551 and 1297, respectively.

### Processing of scRNA-seq Data

The raw scRNA-seq data was processed using the 10X Genomics Cell Ranger software (v5.0.1). The CellRanger mkfastq command was used to generate Fastq files. Subsequently, sequencing data were mapped to the pre-build mouse reference genome (mm10, version 1.2.0). The mapping rate of the reads mapped to the genome for the three samples was more than 93%. The CellRanger count command was run on the individual Fastq datasets to produce expression data. The Cellranger aggr program was run to generate the aggregate dataset.

### scRNA-seq Data Analysis

After aggregation of the three samples, R package Seurat v4.0 was used for cell filtration, normalization, principal component analysis, variable genes finding, clustering analysis, and UMAP dimensional reduction. The datasets were trimmed by removing the cells expressing fewer than 200 genes and the genes expressed in fewer than five cells. Cells with an expression of >3000 genes were filtered out for exclusion of possible cell aggregates. Cells containing >10% mitochondrial genes were presumed to be of poor quality and were also filtered out. Data were then log-normalized for the subsequent analyses. Principal component analysis (PCA) was used for dimension reduction, followed by clustering analysis in principal component analysis space using a graph-based clustering approach. UMAP was then used for the two-dimensional visualization of the identified clusters. The Seurat functions DotPlot, Vlnplot, FeaturePlot, and Heatmap were used to visualize the gene expression with dot plot, violin plot, feature plot, and heatmap, respectively. Markers for a specific cluster against all remaining cells were identified by using the Seurat function FindAllMarkers.

### Statistical Analysis

The data corresponding to mouse body weight, plasma glucose, total cholesterol, and triglyceride were presented as mean ± standard error of the mean. Statistical analysis was performed using GraphPad Prism 8.0 Software (GraphPad Software, San Diego, CA, United States). All data were tested for normality and similar variance. Then, unpaired *t*-test was used to compare the body weight, plasma total cholesterol, and triglyceride. Two-way ANOVA followed by Bonferroni test was used to compare the plasma glucose concentration between Chow and DDC groups at the indicated time.

## Data Availability Statement

The datasets presented in this study can be found in online repositories. The names of the repository/repositories and accession number(s) can be found below: https://www.ncbi.nlm.nih.gov/geo/, GSE169332.

## Ethics Statement

The animal study was reviewed and approved by the Institutional Animal Care & Use Committee (IACUC) from the University of Michigan.

## Author Contributions

GZ, HL, YL, and YZ performed the experiments. GZ performed data analysis. GZ and JZ wrote the article. TZ and MG-B provided technical support and contributed to the discussion of the article. MG-B did critical editing. YEC, JZ, and GZ designed research and discussed the results. All authors contributed to the article and approved the submitted version.

## Conflict of Interest

The authors declare that the research was conducted in the absence of any commercial or financial relationships that could be construed as a potential conflict of interest.
