# Supplementary material for: Single-Cell Transcriptomics Reveals Endothelial Plasticity During Diabetic Atherogenesis
Source: Front Cell Dev Biol. 2021 May 19;9:689469. doi: 10.3389/fcell.2021.689469 (PMC8170046; doi:10.3389/fcell.2021.689469)
Supplement: Supplementary file 2 [file Data_Sheet_2.docx]

Supplementary Material

Single Cell Transcriptomics Reveals Endothelial Plasticity During Diabetic Atherosclerosis

**Guizhen Zhao^1^, Haocheng Lu^1^, Yuhao Liu^1, 2^, Yang Zhao^1^, Tianqing Zhu^1^, Minerva T. Garcia-Barrio^1^, Y. Eugene Chen^1^*****, Jifeng Zhang^1^***

^1^ Frankel Cardiovascular Center, Department of Internal Medicine, University of Michigan Medical Center, Ann Arbor, MI 48109

^2^ Department of Internal Medicine, the Second Xiangya Hospital, Central South University, Changsha, 410011, P. R. China

***** Corresponding Authors:

Jifeng Zhang, PhD. Email: [jifengz@umich.edu](mailto:mailto:jifengz@umich.edu)

Y. Eugene Chen, MD, PhD. Email: [echenum@umich.edu](mailto:mailto:echenum@umich.edu)

# Supplementary Figures

**
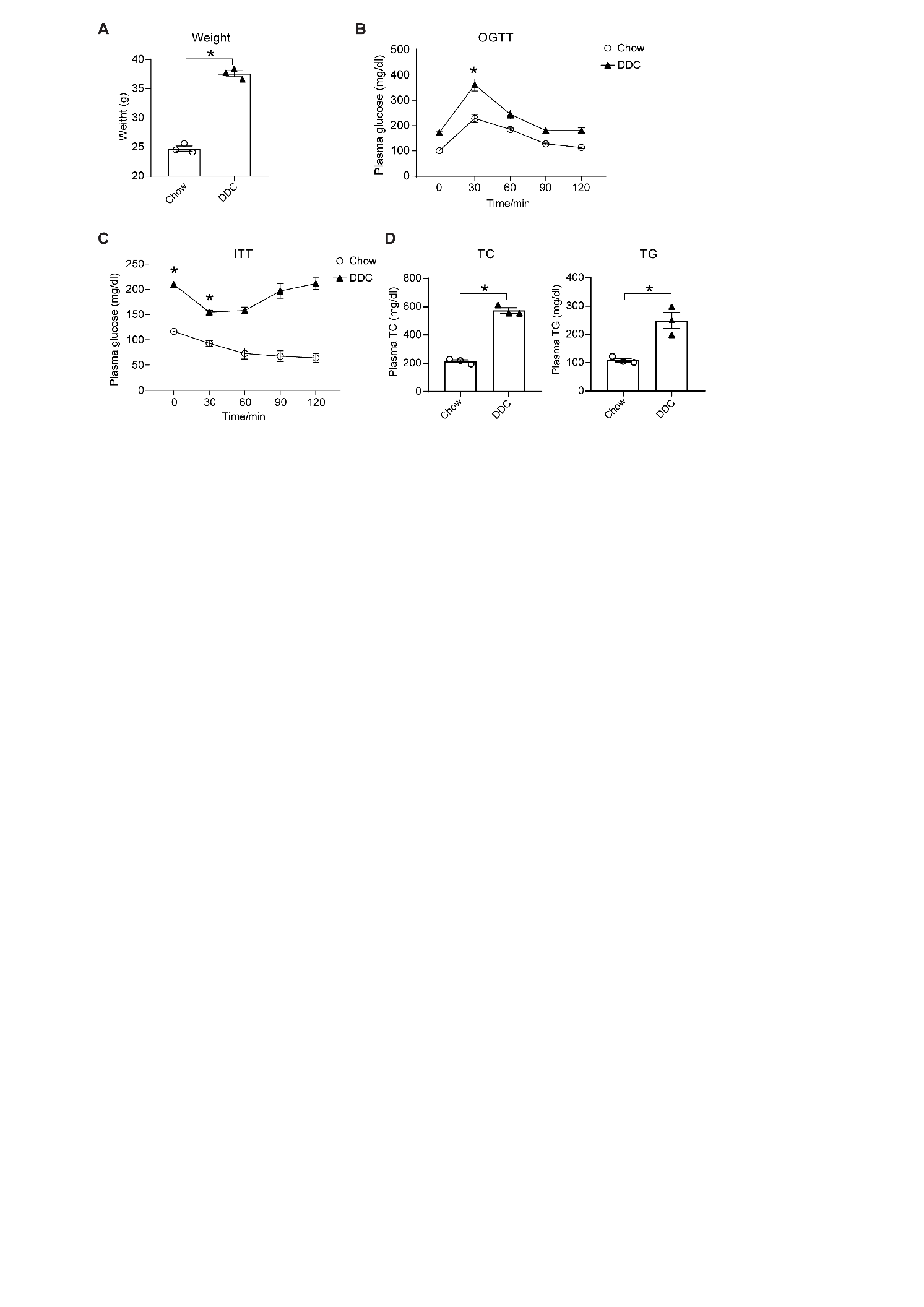
**

**Supplementary Fig.1. Diabetogenic diet plus cholesterol induces obesity, glucose intolerance, insulin resistance and hyperlipidemia in *Ldlr*^-/-^ mice.** Eight-week-old male *Ldlr^-/-^* mice were fed standard chow or a diabetogenic diet with 0.15% cholesterol for 12 weeks. N=3 for each group. **A**, Body weight in the chow- or DDC-fed *Ldlr^-/-^* mice. **B**, Oral glucose tolerance test (OGTT) following 12 hours of fasting was determined in *Ldlr^-/-^* mice fed Chow or DDC for 12 weeks. **C**, Insulin tolerance test following 6 hours of fasting and injection of insulin intraperitoneally (1 U insulin/kg) in *Ldlr^-/-^* mice fed Chow or DDC for 12 weeks. **D**, Plasma total cholesterol (TC) and triglyceride (TG) levels in Chow- or DDC-fed *Ldlr^-/-^* mice. Data are presented as mean±SEM. Unparied t-test for A and D. Two-way ANOVA followed by Bonferroni test for B-C. ^*^*P*<0.05.

**
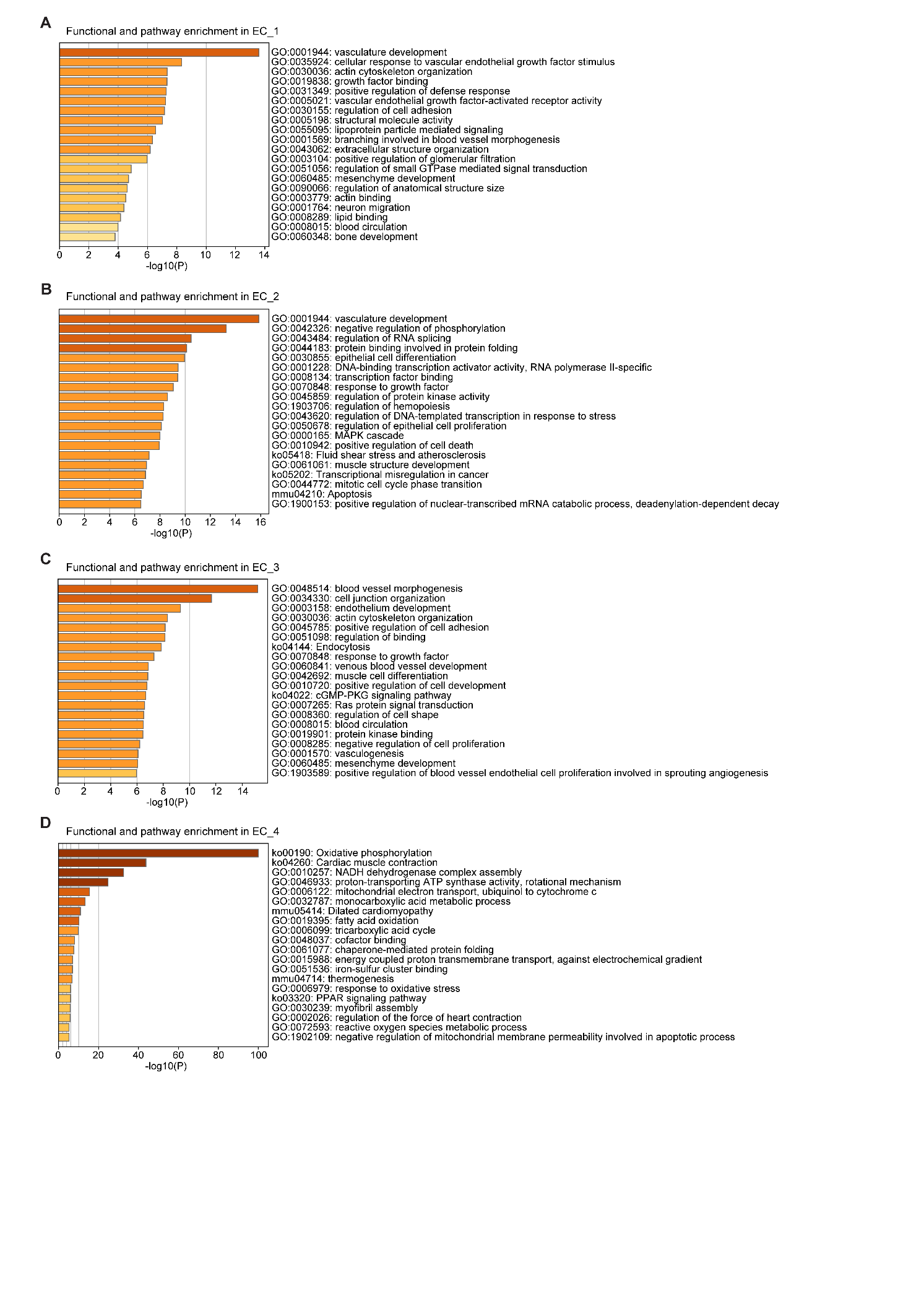
**

**Supplementary Fig.2. A-D.** Functional and pathway enrichment analysis of EC clusters 1 to 4. The top 20 enriched pathways and biological processes are shown for each EC cluster.

**
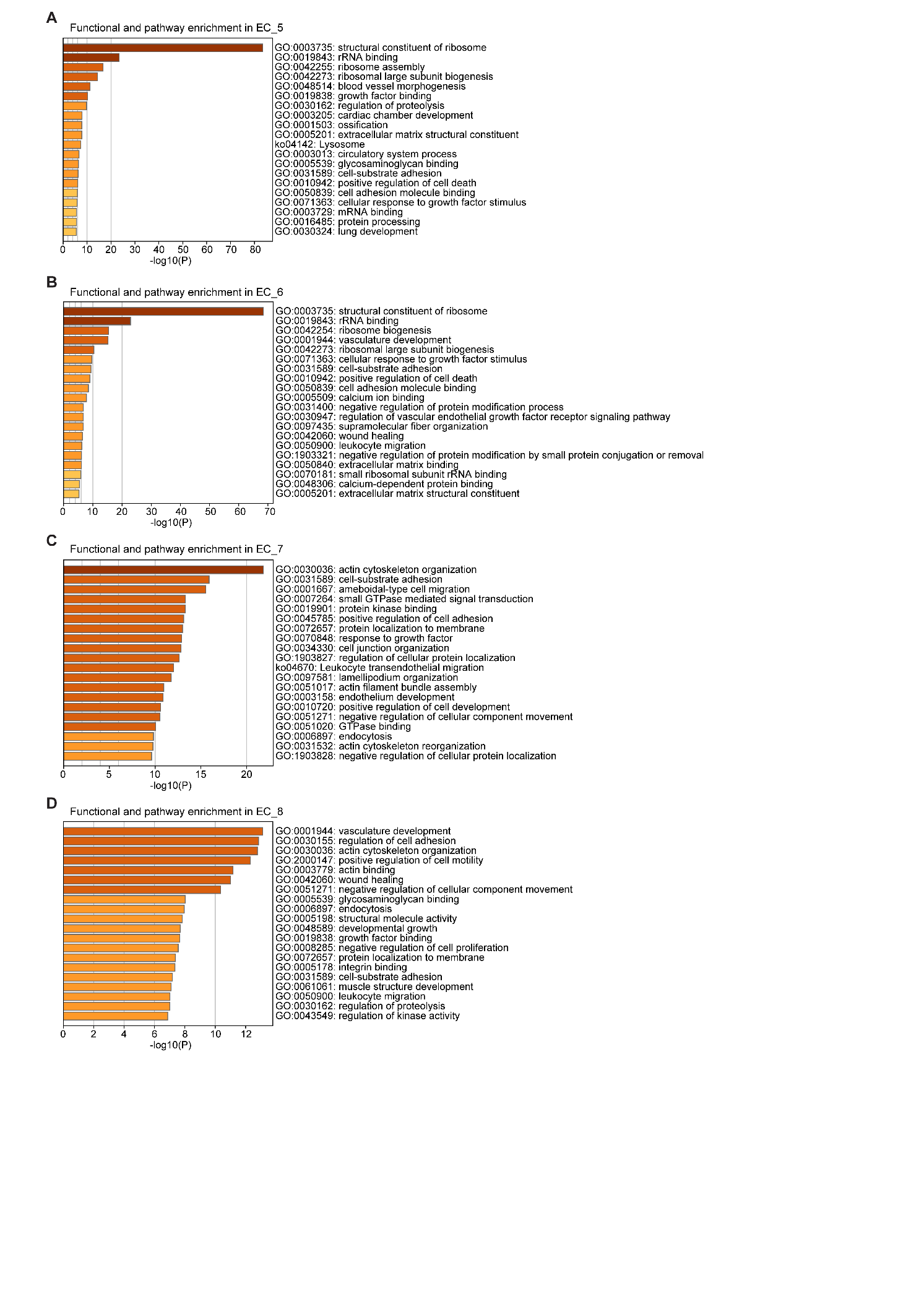
**

**Supplementary Fig.3. A-D.** Functional and pathway enrichment analysis of EC clusters 5 to 8. The top 20 enriched pathways and biological processes are shown for each EC cluster.


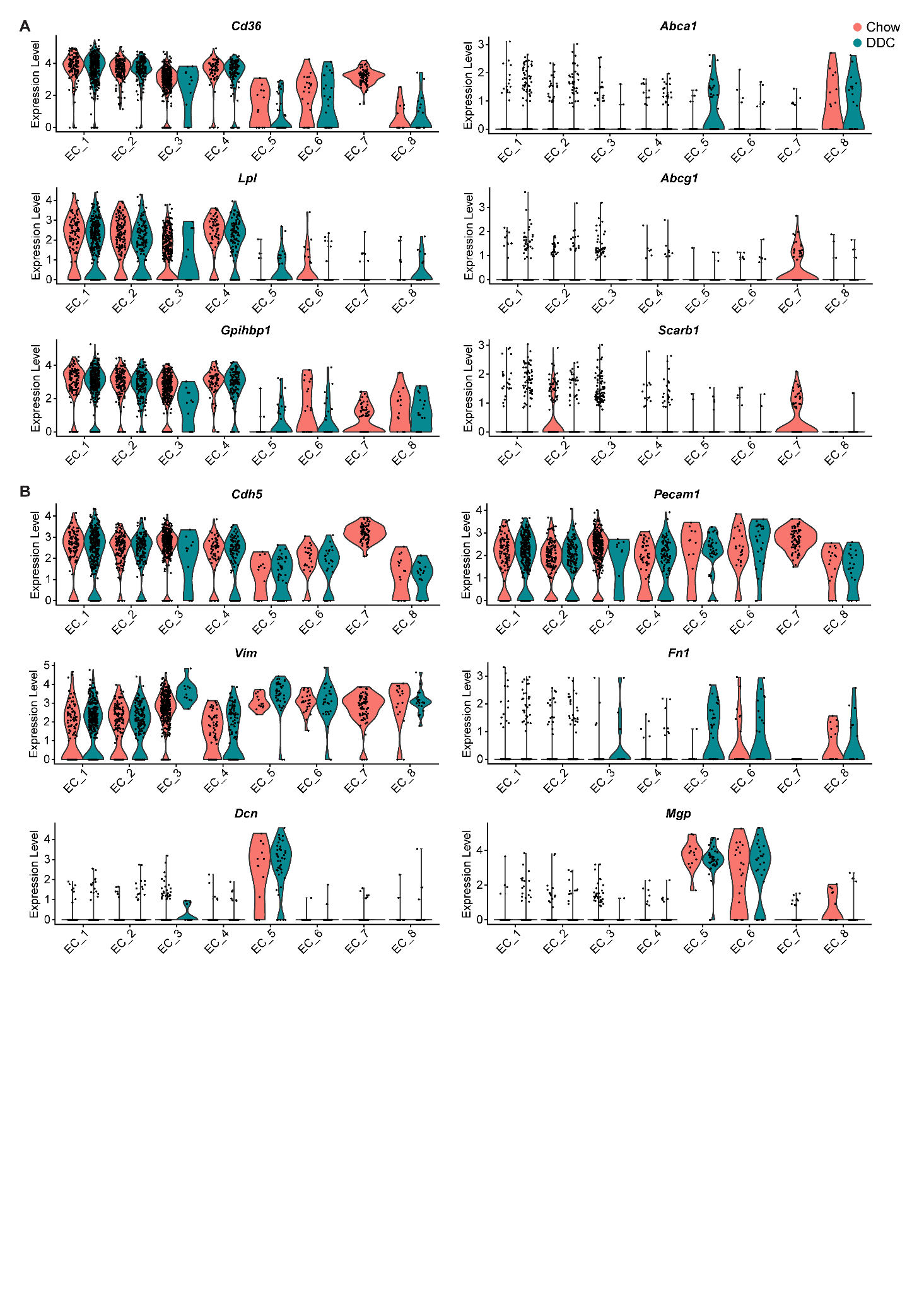


**Supplementary Fig.4.A-B.** Violin plots demonstrating the expression levels of the lipid-handling genes (*Cd36*, *Lpl*, *Gpihbp1*, *Abca1*, *Abcg1*, *Scarb1*), canonical EC marker genes (*Cdh5*, *Pecam1*), and mesenchymal marker genes (*Vim*, *Fn1*, *Dcn*, *Mgp*) in each EC cluster with the color denoting experimental conditions and each dot indicating an individual cell.
